# Evaluation of the Fracture Behavior of Severely Damaged Endodontically Treated Anterior Teeth with Different Core Systems

**DOI:** 10.4317/jced.62936

**Published:** 2025-11-30

**Authors:** Marine Ortiz-Magdaleno, Marlene Castillo-Sanay, Norma Verónica Zavala-Alónso, José Manuel Gutiérrez-Hernández

**Affiliations:** 1 Specialty in Aesthetic, Cosmetic, Restorative, and Implant Dentistry, Faculty of Stomatology, Autonomous University of San Luis Potosi, San Luis Potosi, Mexico; 2 Department of Dental Sciences Advanced Education, Faculty of Stomatology, Autonomous University of San Luis Potosi, San Luis Potosi, Mexico; 3 Basic Sciences Laboratory, Faculty of Stomatology, Autonomous University of San Luis Potosi, San Luis Potosi, Mexico

## Abstract

**Background:**

The purpose of this in vitro study was to determine the fracture behavior of severely damaged anterior endodontically treated teeth with a core buildup and polyethylene ribbon fiber in different orientations.

**Material and Methods:**

Forty human maxillary incisors that had been endodontically treated were prepared to receive a core buildup and divided into 4 groups (n=10): glass fiber post, polyethylene ribbon fiber inserted horizontally, polyethylene ribbon fiber inserted vertically, and core only. The cores were made with short fiber resin composite. The teeth were statically loaded on the palatal surface until fracture, and the failure mode was classified. The mean values of the groups were analyzed by using the Shapiro-Wilk test and 1-way analysis of variance (=.05).

**Results:**

The highest fracture loads were observed in the polyethylene ribbon groups: vertical insertion (677.6 ± 2.4 N) and horizontal insertion (657.1 ± 2.2 N). These were followed by the glass fiber post (421.5 ± 2.2 N) and the core-only group (246.3 ± 4.0 N). The polyethylene ribbon fiber groups were statistically similar (p&gt;0.05). The greatest mean maximum strain, stress, and fracture strength were obtained by the core buildup with polyethylene ribbon fiber inserted horizontally. No significant difference in the fracture strength and maximum stress was found among the glass fiber post, core only, and polyethylene ribbon fiber groups inserted vertically groups (p&gt;0.05). The ratio of catastrophic to non-catastrophic failure was similar for the polyethylene ribbon fiber groups (both 50% - 50%) and for the glass-fiber post (60% - 40%) and core only (70% - 30%).

**Conclusions:**

Cores made with short fiber resin composite with polyethylene ribbon fiber inserted vertically or horizontally can be used as an alternative to conventional post systems.

## Introduction

Different treatments are available for restoring severely damaged endodontically treated anterior teeth (1). Restorations using polyethylene ribbon fibers and short fiber resin composite provide good adaptation in the intracanal post and reinforcement of the remaining dental tissue, but which direction of the polyethylene ribbon fiber promotes higher values of fracture resistance is not clear (2 , 3). The number and orientation of the segments of polyethylene ribbon fiber have been reported to influence the mechanical behavior of severely damaged anterior endodontically treated teeth (4). A widely used technique to enhance fracture strength and resistance involves placing one segment of polyethylene ribbon fiber vertically or two segments horizontally using a short fiber resin composite as the core and post material (5 , 6). A core buildup replaces missing coronal structure in an endodontically treated tooth using different restorative procedures and materials, supporting the crown. The core maintains the integrity of the tooth, strengthening the tooth structure and decreasing the risk of fracture and failure. Factors that affect the prognosis for severely damaged anterior endodontically treated teeth include the remaining healthy tooth structure, the functional requirements of the tooth, and esthetics (7 - 9). When the tooth has insufficient remaining coronal structure, an intracanal post, such as a glass fiber post, is often placed to provide retention for-up and support for a crown. However, the removal of sound tissue during the post-space preparation should be avoided, and an excessive post space may be associated with vertical root fracture (10 , 11). Glass fiber post failure sare often linked to poor fit due to irregular root canal geometry and flaring in the cervical third of anterior teeth (12). Short fiber-reinforced resin composites have been reported to be suitable material for a core within the root canal as they act as a bioblock and have physical properties similar to those of dentin. The material can also be reinforced with polyethylene ribbon fiber as its flexibility permits adaptation to the root canal walls (13) and provides a post material that can stop the propagation of cracks in the restoration (14 - 16). The combination of short fiber-reinforced resin composites with polyethylene ribbon fiber has been reported to improve load capacity because of its mechanical properties and its ability to absorb or dissipate the forces along the root of severely damaged endodontically treated anterior teeth, thereby reducing stresses during mastication (17 - 21). A standard protocol for the treatment of severely damaged endodontically treated anterior teeth is lacking, and which buildup method and post-and-core system provides the best clinical performance is not clear. Studies on the effect of different combinations of restorative materials (resin composites, glass fiber post, short fiber-reinforced resin composite, polyethylene ribbon fiber, and anatomic post) are needed to determine the influence of fracture resistance and fracture pattern. Therefore, the aim of this in vitro study was to evaluate the fracture behavior (maximum load, fracture strength, fracture resistance, and maximum stresses) of severely damaged endodontically treated anterior teeth by reinforcing the core buildup with polyethylene ribbon fiber in 2 different orientations (vertically or horizontal) versus glass fiber posts and core buildup-up with only short fiber- reinforced resin composite without a glass fiber post. The null hypothesis was that no difference in fracture behavior would be found between the different adhesive restorative methods.

## Material and Methods

Fourteen intact and caries-free extracted human maxillary central incisors teeth with similar sizes and root dimensions were obtained under the approval of the Ethical Committee of the Faculty of Stomatology (CEI-FE-011022). They were cleaned of debris and soft tissue remnants and stored in physiological saline at 4 ºC for up to 3 months until use. The clinical crowns of all teeth were removed at the cement-enamel junction (CEJ) perpendicularly to the long axis of the tooth using a smooth silicon carbide cutting disk with constant water-cooling irrigation and a low-speed precision cutter machine (IsoMet 5000; Buehler). All the root lengths were adjusted to 15 mm from the root apex and were coated with a 0.2-mm layer of polytetrafluoroethylene to simulate the periodontal ligament before being embedded in acrylic resin (Nic Tone; MDC Dental) with the long axis of the tooth perpendicular to the base of the block up to 3 mm apical to the CEJ to simulate the alveolar bone. Then, with a conical round bur, the ferrule area was prepared in the dentin to a height of 2.5 mm around each tooth. The sample size was determined using statistical power analysis software (G*Power 3.1.9.3; Heinrich-Heine-Universität Düsseldorf). The effect size was hypothesized to be 0.57. Accordingly, with .05 significance level and .8 power, the projected sample size needed was 10 specimens per group. Endodontic treatment was performed, and standardized endodontic access cavities were prepared by a single operator (M.C.) according to the procedure described previously. After endodontic treatment, the access cavities were cleaned and dried, and the pulp chamber floor was layered with a 1-mm-thick light-polymerizing reinforced glass ionomer restorative material (II GC Universal Restorative; GC Corp). The remainder of the endodontic access cavity was then initially filled with a light-polymerizing composite resin (Systemp Inlay; Ivoclar AG). The teeth were stored at 37ºC and 100% humidity for 7 days to ensure that the resin had fully polymerized. Table 1 shows the materials used in the present study.

Table 1

**Figure 1:**
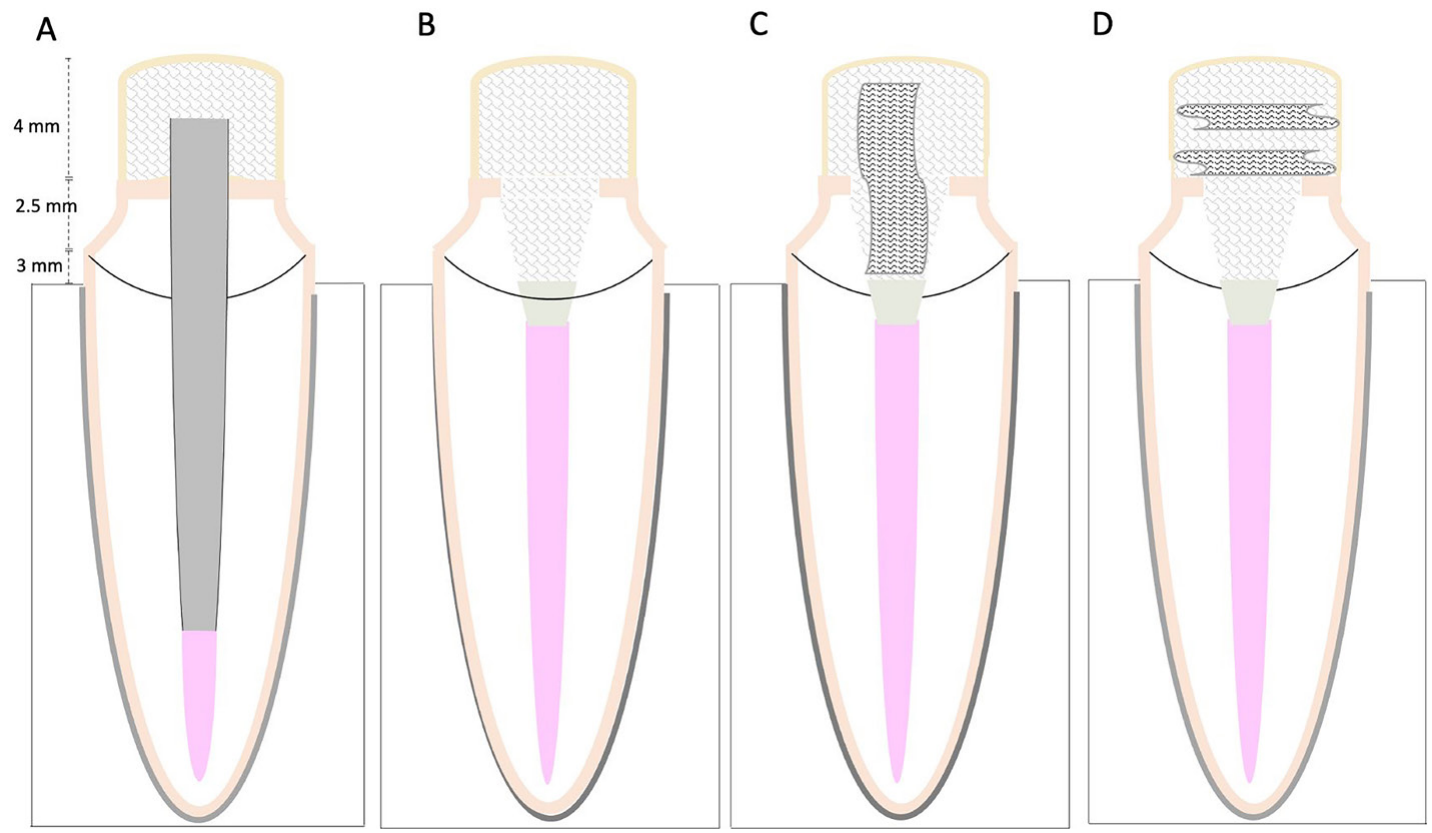
Schematic representing experimental groups with different adhesive restorative methods for core buildup treatments. A, Glass fiber post. B, Short fiber-reinforced resin composite. C, Polyethylene ribbon fiber inserted vertically. D, Polyethylene ribbon fiber inserted horizontally.

Forty roots (n=40) were randomly divided into four subgroups (n=10) by drawing lots according to the restored material for the core build-up (Fig. 1).

1

**Figure 2:**
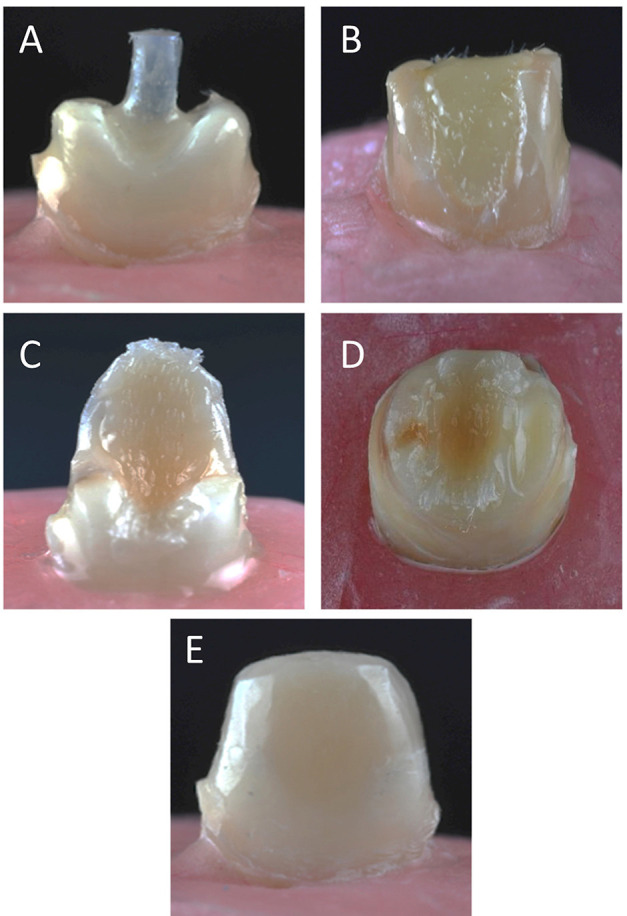
Images of specimens during core placement A, Glass fiber post. B, Short fiber-reinforced resin composite. C, Polyethylene ribbon fiber inserted vertically. D, Polyethylene ribbon fiber inserted horizontally. E, Core buildup with layer of resin composite.

The first group were teeth restored with a glass fiber post (RelyX Fiber Post; 3M ESPE); the coronal and middle thirds of the root canal were prepared with a #4 Gates-Glidden drill (Dentsply Sirona) under water cooling, leaving 3 to 5 mm of the apical gutta percha. The root canals were then sequentially enlarged to 1.5 mm. This procedure standardized the post lengths (RelyX Fiber Post; 3M ESPE) and diameters in this group. The post surface was cleaned with 95% ethanol and cemented with a resin cement (RelyX U200; 3M ESPE) manipulated according to the manufacturer's instructions. The posts were coated with cement and inserted with light hand pressure, and, after the post reached the regulated length, any excess luting material was removed with an explorer. A polymerization unit with a Ø8-mm light tip and 1400 mW/cm2 power (Bluephase; Ivoclar AG) was placed in contact with and orthogonal to the post and activated for 40 seconds. In all the groups, the remaining coronal structure was first etched for 15 seconds using a 35% phosphoric acid (Scotchbond Etchant; 3M ESPE), washed with water spray for 15 seconds, and gently air dried for 5 seconds. Dentin primer (Scotchbond Multi-Purpose Primer; 3M ESPE) was applied, lightly air dried for 5 seconds, and then light polymerized for 10 seconds, as suggested by the manufacturer. The same adhesive treatment was used for all teeth. The core buildup was formed with 2-mm layers of short fiber resin composite, and a 6-mm length of polyethylene ribbon fiber was placed in a vertical orientation, a 3-mm length was placed within the root canal, and a 3-mm length of the polyethylene ribbon fiber was placed outside of the canal to serve as the core; the short fiber resin composite was condensed inside the root canal and light polymerized for 40 seconds (Fig. 2).

2

**Figure 3:**
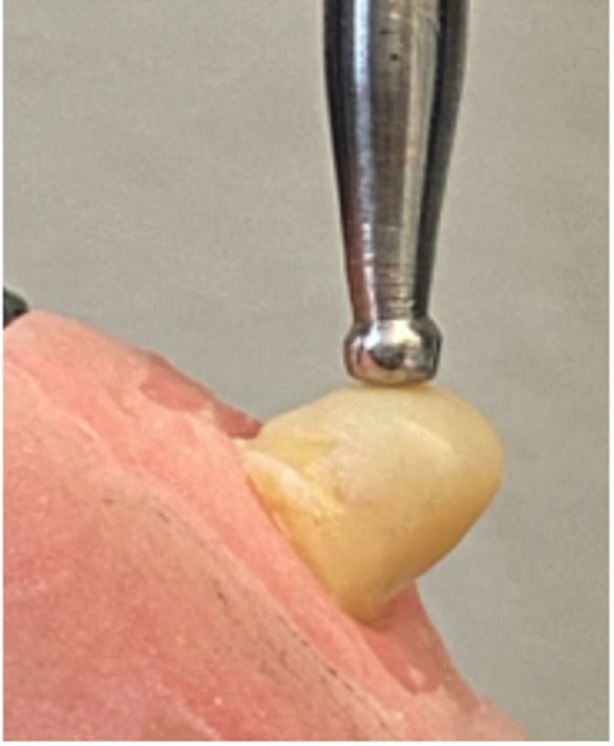
Specimen in acrylic resin block in universal testing machine for fracture strength testing.

For the group buildup with horizontal polyethylene ribbon fiber, 2 separate 6-mm lengths of polyethylene ribbon fiber were placed under the short fiber resin composite increments in a labio-palatal direction and light polymerized. The polyethylene ribbon fibers were impregnated with resin before application. To have the same core dimensions, a transparent template matrix (Elite transparent; Zhermack) of a well-constructed core was used to form the cores with 6 mm of buildup from the incisal to the finish line lingually and labially and with 7 mm from mesial to distal. All the cores were restored with short fiber resin composite in increments of 2 mm, and each layer was light polymerized for 40 seconds. The last layer was added with resin-based composite (Clearfil AP-X ES-2; Kuraray Noritake Dental) to cover the core. All cores were placed according to the manufacturer's instructions, finished using a fine-grit diamond rotary instrument, polished with aluminum oxide disks (Sof-Lex, Pop On; 3M ESPE), and stored at 37°C for 1 week. The acrylic resin block containing the restored tooth was tightly fixed to the inclined metal base to provide a 45-degree angle between the palatal surface of the tooth and the Ø2-mm spherical loading tip in the middle third. A static load was applied to the crowns 2-mm apical to the incisal edge on the palatal side at a 45-degree angle until fracture using a universal testing machine (Shimadzu PWS-E100) with a crosshead speed of 1 mm/minute (Fig. 3).

3

The fracture resistance was recorded in N. The fracture strength in MPa was calculated by dividing the fracture resistance by the bonding surface area (42 mm2). The mode of failure after fracture for each specimen was visually analyzed by an external investigator and categorized into non-catastrophic or catastrophic failure. Statistical analysis was done using a statistical software program (Sigma Plot version 11.0; Inpixon). The normality of the data distribution was determined with the Shapiro-Wilk test. Since the data followed a normal distribution, the data were analyzed by 1-way analysis of variance (ANOVA) and pairwise comparisons with the Tukey test (=.05).

## Results

**Figure 4:**
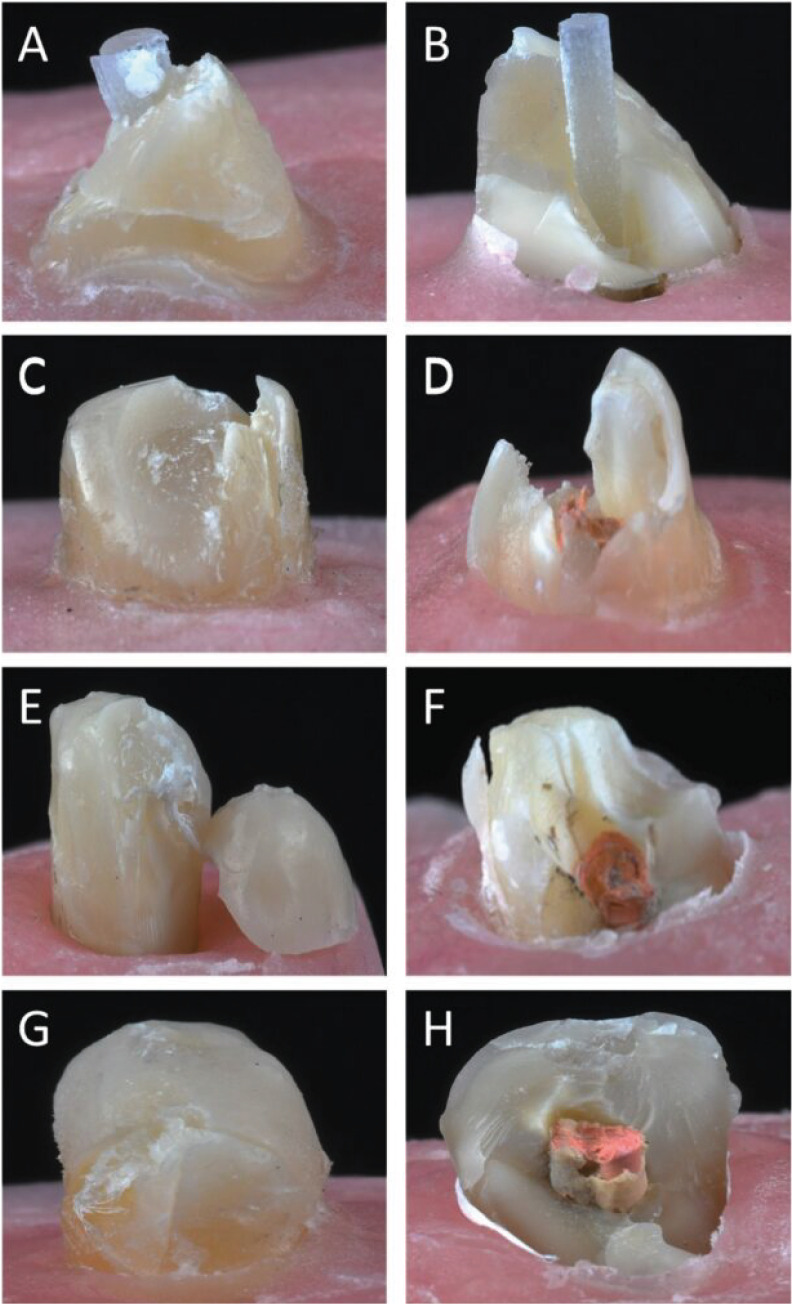
Photographs of failure modes of tested restorations. Non-catastrophic and catastrophic mode respectively. A, B, Glass fiber post. C, D Short fiber-reinforced resin composite. E, F, Polyethylene ribbon fiber inserted vertically. G, H, Polyethylene ribbon fiber inserted horizontally.

Table 2 presents the values of the 4 groups and Figure 4 shows the failure modes and the percentage of each group.

Table 2

4

The highest maximum strain values were recorded in the polyethylene ribbon fiber inserted horizontally, followed by the polyethylene ribbon fiber inserted vertically and the glass fiber post group; the lowest value was obtained by the short fiber resin composite core only. One-way ANOVA revealed significant differences among core buildup groups. The horizontally inserted polyethylene ribbon fiber group showed statistically significant differences compared to the vertically inserted polyethylene ribbon fiber, glass fiber post, and core-only groups( p0.011). The polyethylene ribbon fiber inserted horizontally was statistically similar to the polyethylene ribbon fiber inserted vertically and the glass fiber post (p&gt;.05); the short fiber resin composite core only showed statistically significant differences from polyethylene ribbon fiber inserted horizontally or vertically and the glass fiber post (p.012). The fracture strength of the restored teeth revealed that the buildup technique with polyethylene ribbon fiber inserted horizontally showed the highest values, which were statistically significantly different from those of polyethylene ribbon fiber inserted vertically, the short fiber resin composite core only, and the glass fiber post (p.003). The groups restored with polyethylene ribbon fiber inserted vertically, the short fiber resin composite core only, and the glass fiber post were statistically similar. The polyethylene ribbon fiber inserted horizontally and vertically exhibited high and statistically similar (p&gt;.05) fracture resistance, followed by the glass fiber post and short fiber resin composite core. The lowest fracture resistance values were recorded in the short fiber resin composite core only group, which showed statistically significant differences from glass fiber post (p=.003). The ANOVA showed that cores reinforced by polyethylene ribbon fiber inserted horizontally had a statistically significantly higher maximum stress than polyethylene ribbon fiber inserted vertically polyethylene ribbon fiber inserted vertically, glass fiber posts, and short fiber resin composite cores (p.003). The maximum stress in the groups with polyethylene ribbon fiber inserted vertically, short fiber resin composite cores and glass fiber posts were statistically similar (p&gt;.05). The buildups with polyethylene ribbon fiber inserted horizontally and vertically had 50% catastrophic and 50% non-catastrophic failures. The group restored with glass fiber posts had a predominance of catastrophic failures (60%), with 40% non-catastrophic; the short fiber resin composite cores had 30% catastrophic failures and 70% not catastrophic failures.

## Discussion

Mechanical parameters, the maximum load, fracture strength, fracture resistance, and maximum stress were evaluated to compare restorations with different core systems in severely damaged endodontically treated anterior teeth. Polyethylene ribbon fiber was placed in 2 different orientations, to identify which restorative protocol demonstrated better mechanical behavior or, how the type of core restorative material and restorative procedure affected the load-bearing capacity in severely damaged endodontically treated anterior teeth. The study demonstrated that horizontally and vertically increased polyethylene ribbon fiber significantly improved fracture strength and resistance in severely damaged endodontically treated anterior teeth and exhibited 50% of restorable fractures and 50% of non-restorable fractures. Therefore, a direct relationship between the orientation and the number of segments of polyethylene ribbon fiber affected the tooth fracture strength. Moreover, the cores restored with the polyethylene ribbon fiber inserted into the full depth of the prepared spaces made root canal preparation for a glass fiber post unnecessary. The combination of short fiber resin composite and polyethylene ribbon fiber acts as bondable reinforcement that absorbs stresses and internally splints the core with the severely damaged endodontically treated anterior tooth, thus distributing forces in more than one direction and providing retention for the core, which supports the crown. Alternative methods of restoring endodontically treated anterior teeth have included accessory posts in addition to the main glass fiber post, glass fiber posts relined with resin composite according to the anatomy of the root canal, and glass fiber posts reinforced with polyethylene ribbon fiber to fill the space between the post and the dentin root canal walls, this with the objective of reducing the cement film thickness in the cervical third (22 , 23). This in vitro study showed that the severely damaged endodontically treated anterior teeth with a core buildup with polyethylene ribbon fiber oriented horizontally and vertically had higher values of maximum strain, fracture strength, fracture resistance and maximum stress than the groups with core buildups with glass fiber posts or short fiber resin composite. Therefore, the null hypothesis that no difference in fracture behavior would be found between the different adhesive restorative methods was rejected. The mechanical properties obtained with the insertion of short fiber resin composite and a glass fiber post was lower than the cores reinforced with polyethylene ribbon fiber. Therefore, placing polyethylene ribbon fiber in a vertical or horizontal orientation instead of a glass fiber post produces the necessary mechanical properties to replace dentin in severely damaged endodontically treated anterior teeth. The short fiber resin composite cores had fracture strength and resistance values below those of the polyethylene ribbon fiber inserted horizontally and vertically groups, 70% of non-catastrophic failures, and 30% of catastrophic failures. However, the percentage of non-catastrophic failures was the highest among the other groups. Regarding the group of cores with glass fiber posts, the fracture strength and fracture resistance values were the lowest, but the values of maximum strain were close to those of the glass fiber posts group. In this study, the effect of different resin core systems on fracture resistance has been evaluated. However, because of the use of different parameters in the assays and materials used, a comparison of the restorative protocols is difficult. Kubo et al. (21) recommended a glass fiber post combined with polyethylene ribbon fiber for structurally compromised teeth with flared root canals. They reported significantly higher fracture strength with a glass fiber post combined with polyethylene ribbon fiber and with only polyethylene ribbon fiber versus the glass fiber post control group. Priyanka et al. (22) compared the fracture resistance of endodontically treated maxillary anterior teeth using glass fiber posts reinforced with polyethylene ribbon fiber, anatomic glass fiber posts, and glass fiber posts luted with a dual-polymerizing cement and concluded that the use of anatomic glass fiber posts in wide and flared canals provided the highest fracture resistance. Placing a glass fiber post has been one of the most used restorative strategies for severely damaged endodontically treated anterior tooth, as it absorbs the masticatory forces and distributes them along the root, as well as retaining the core material (23). However, the insertion of glass fiber posts has been reported to be potentially prejudicial because their shape does not match the morphology of the root canal, resulting in a higher number of unfavorable failures (24 , 25). Because of their high compressive and fracture strength values, in conjunction with their low polymerization shrinkage, short fiber resin composites and polyethylene ribbon fiber have been used for different applications in restorative dentistry, including to strengthen structurally compromised teeth so that they can withstand the masticatory load better than resin composite restorations (25). However, studies on which orientation of the polyethylene ribbon fiber better increases fracture strength and fracture resistance values are lacking. Behzadpour et al. (26) studied the fracture strength and fracture mode of primary canine teeth reconstructed with composite resin posts, glass fiber posts, custom glass fiber posts with Interlig fibers, or custom polyethylene ribbon fiber. However, in their study, the tooth crown was restored with bulk-fill composite resin, and the fracture strength was significantly lower in the resin composite group than in the other groups with polyethylene ribbon fiber. In the present study, only the load on the crowns was evaluated, as the main objective was to determine which of the restorative protocols showed the best mechanical behavior. Alamdari et al. (27) compared the fracture resistance of severely damaged primary anterior teeth with glass fiber posts and short fiber resin composite and concluded that this system enhanced their fracture resistance and increased the chance of reparability in case of restoration fracture. Moreover, it is also in agreement with the results of our study that fracture resistance was significantly higher using glass fiber posts and short fiber resin composite than that only short fiber resin composite. Albashaireh et al. (28) reported that the use of polyethylene ribbon fiber to reinforce resin composite cores significantly increased fracture resistance in endodontically treated anterior teeth compared with the use of glass fiber posts with direct resin composites. The results in the present study demonstrated that the teeth restored with glass fiber posts and short fiber resin composite had higher values of maximum strain, fracture resistance, and maximum stress compared with the cores with short fiber resin composite, except for fracture strength. Although, the cores with only short fiber resin composite had a lower incidence of catastrophic failure, they had the lowest values of maximum strain, fracture resistance, and maximum stress. Limitations of this study included that the anterior endodontic teeth were not provided with crowns or stressed with artificial aging and cyclic loading, simulating clinical use in the oral cavity. The efficacy of these techniques should be evaluated in longitudinal clinical studies.

## Conclusions

Based on the findings of this in vitro study, the following conclusions were drawn: 1. Cores reinforced with vertically or horizontally oriented polyethylene ribbon fiber and short fiber resin composite exhibited greater fracture resistance and strength, likely due to enhanced stress distribution. The failure mode was not significantly different between the 2 groups. 2. A statistically significant difference was found between the cores with polyethylene ribbon fiber and with glass fiber posts. The values of fracture strength resistance were lower for glass fiber posts, and the non-repairable failure mode was dominant in this group. 3. The cores built up with only short fiber resin composite had the lowest values for the mechanical parameters evaluated, but showed 70% of non-catastrophic failures.

## Figures and Tables

**Table 1:** Materials used.

Brand	Type	Manufacturer	Composition
RelyX Fiber Post	Fiber post	3M ESPE	S-glass fibers (60%–70% by weight).
RelyX U200	Self-adhesive dualpolymerized resin lutingcement	3M ESPE	Base paste: methacrylate monomers containing phosphoric acid groups, methacrylate monomers, silanated fillers, initiator components, stabilizers, rheological additives.
Vitremer	Resin-modified glass ionomercement	3M ESPE	Powder: fluoraluminosilicate glass, redox catalyst system, pigments.Liquid: aqueous solution of polycarboxylic acid modified with pedant methacrylate groups, Vitrebond copolymer, water, HEMA, photoinitiators.Primer: Vitrebond copolymer, HEMA, ethanol, photoinitiators.
Ribbond fibers	Polyethylene ribbon fibers	Ribbond	Preimpregnated, silanized, plasma-treated, leno-woven, ultrahigh–modulus polyethylene fibers.
EverX posterior	Short fiber-reinforced resin composite	GC	Bis-GMA, PMMA, TEGDMA, millimetre scale glass fiber filler, Barium glass 76 wt%, 57 vol%
Clearfill AP-X ES-2	Nano-hybrid resin composite	Kurakay	TEGDMA, Bis-GMA, silanated barium glass, silanated colloidal silica, silanated silica, others (filler by weight 86%, volume 70%; mean particles size 1–3 μm).

Bis-GMA, bisphenol-A-glycidyl dimethacrylate; HEMA, 2-hydroxyethyl methacrylate; PMMA, polymethyl methacrylate; TEGDMA, triethyleneglycol dimethacrylate.

**Table 2:** Mean values and standard deviations (SD) of maximum strain, fracture strength, fracture resistance and maximum stress. Mean difference significant at p<.05.

Experimental groups	Maximum strain (N)	Fracture strength (MPa)	Fracture resistance (N)	Maximum stress (MPa)
Glass fiber post	608.4 ±1.2	14.4 ±2.9	421.5 ±2.2	12.4 ±3.9
Short fiber-reinforced resin composite	147.4 ±3.2	16.2 ±3.9	246.3 ±4.0	10.0 ±2.0
Polyethylene ribbon fiber inserted vertically	683.0 ±1.9	20.0 ±3.4	677.6 ±2.4	14.9 ±3.7
Polyethylene ribbon fiber inserted horizontally	1137.7 ±2.4	29.2 ±4.0	657.1 ±2.2	22.3 ±4.7

2

## Data Availability

The datasets used and/or analyzed during the current study are available from the corresponding author.
